# A meta‐analysis and cost‐minimization analysis of cryoballoon ablation versus radiofrequency ablation for paroxysmal atrial fibrillation

**DOI:** 10.1002/joa3.13055

**Published:** 2024-06-09

**Authors:** Yoshimi Nitta, Michiko Nishimura, Hidetoshi Shibahara, Teiichi Yamane

**Affiliations:** ^1^ Health Economics & Reimbursement Japan Abbott Medical Japan LLC Tokyo Japan; ^2^ CRECON Medical Assessment Inc Tokyo Japan; ^3^ Division of Cardiology, Department of Internal Medicine The Jikei University School of Medicine Tokyo Japan

**Keywords:** atrial fibrillation, catheter ablation, cost‐effectiveness analysis, meta‐analysis, radiofrequency ablation

## Abstract

**Background:**

Previous studies have shown inconsistent results in clinical effectiveness between cryoballoon ablation (CBA) and radiofrequency ablation (RFA), and cost assessment between the procedures is important. The aim of this study was to evaluate the clinical effectiveness and cost‐effectiveness between the procedures in patients with paroxysmal atrial fibrillation (AF) refractory to antiarrhythmic drug therapy.

**Methods:**

A systematic review and meta‐analysis were performed. The primary outcome for the meta‐analysis was long‐term AF recurrence. Following the results of the meta‐analysis, the cost‐effectiveness of CBA versus RFA in Japan was assessed.

**Results:**

The meta‐analysis included 12 randomized controlled trials and six propensity‐score matching cohort studies. AF recurrence was slightly lower in patients referred for CBA than for RFA, with an integrated risk ratio of 0.93 (95% confidence interval: 0.81–1.07) and an integrated hazard ratio of 0.96 (95% confidence interval: 0.77–1.19), but no significant difference was found. A cost‐minimization analysis was conducted to compare the medical costs of CBA versus RFA because there was no significant difference in the risk of AF recurrence between the procedures. The estimated costs for CBA and RFA were JPY 4 858 544 (USD 32 390) and JPY 4 505 255 (USD 30 035), respectively, with cost savings for RFA of JPY 353 289 (USD 2355).

**Conclusion:**

Our meta‐analysis suggests that CBA provides comparable benefits with regard to AF recurrence compared with RFA, as shown in previous studies. Although the choice of treatment should be based on patient and treatment characteristics, RFA was shown that it might be cost saving as compared to CBA.

## Graphical abstract

Our meta‐analysis showed that in patients with paroxysmal atrial fibrillation (AF) refractory to antiarrhythmic drug therapy, cryoballoon ablation (CBA) provides comparable benefits with regard to AF recurrence compared with radiofrequency ablation (RFA). A comparison of the costs of the two procedures showed that RFA might be cost‐saving compared with CBA.

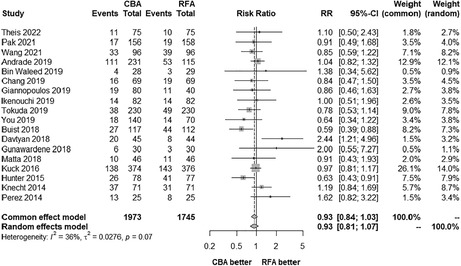

## 1 INTRODUCTION

Atrial fibrillation (AF) is one of the most common types of arrhythmias in clinical practice. In the U.S., the prevalence of AF in the general population increases with advancing age, affecting 2.3% of people aged older than 40 years and 5.9% of people aged older than 65 years. It has been reported that approximately 70% of patients with AF are between 65 and 85 years of age.^1^ In Japan, the number of patients with AF is estimated to be around one million.^2^ AF often starts as episodes of short duration, a condition referred to as paroxysmal atrial fibrillation (PAF). After repeated episodes, AF gradually progresses to non‐self‐terminating persistent and permanent AF.^3^ AF is associated with an increased risk of stroke, heart failure, and death.^4^ Even patients with PAF are known to be at an increased risk of stroke or non‐cerebral embolism.^5^

Catheter ablation is one of the most important treatments for patients with AF. Radiofrequency ablation (RFA), using radio‐frequency as an energy source has been widely accepted as a standard treatment for eliminating AF. With the recent advancements in 3D mapping systems and introduction of contact force (CF)‐sensing, RFA is becoming a more efficient and safer ablation technique.^6^ In recent years, pulmonary vein (PV) isolation using a single‐shot device has also come to be used. Cryoballon ablation (CBA) is the most widely used single‐shot technology that uses nitrous oxide gas to cool surrounding tissues.^6^

The therapeutic effects of the two ablation technologies have been meta‐analyzed or network meta‐analyzed by the National Institute for Health and Care Excellence (NICE), a UK health technology assessment (HTA) body, and in previous studies.^7^, ^8^, ^9^, ^10^, ^11^, ^12^ While many of the studies reported that the risk of AF recurrence after CBA is similar to that after RFA, there is a report to show that CBA is superior to RFA, indicating inconsistent results.^7^ Given that observational studies using propensity score matching method have been conducted in Japan to compare CBA and RFA in recent years, a reassessment incorporating the results from these reports should yield useful findings.^13^, ^14^ Additionally, evaluation of the cost‐effectiveness of these ablation techniques is of great importance given the economic impact of PAF.

Therefore, the objectives of this study were (1) to investigate the clinical effectiveness of treatment with CBA compared with that of RFA in patients with PAF who have previously failed one or more antiarrhythmic drug (AAD) and would receive ablative treatment for the first time for rhythm‐control purposes through a systematic review and a meta‐analysis and (2) to assess the cost‐effectiveness of CBA compared with RFA for these patients in Japan.

## 2 METHODS

### 2.1 Study selection for systematic review and meta‐analysis

This systematic review and meta‐analysis were performed according to the PRISMA (Preferred Reporting Items for Systematic Reviews and Meta‐Analysis) statement for conducting systematic reviews and meta‐analyses of healthcare interventions.^15^

Two reviewers carried out a computerized literature search of MEDLINE, EMBASE, the Cochrane Central Register of Controlled Trials (CENTRAL), and Ichushi‐Web databases from inception until January 2022, to identify relevant studies. Two reviewers then independently read the abstract and full text of the studies according to the specified inclusion and exclusion criteria and evaluated the quality of the studies and extracted the data. In English databases, we combined disease terms (PAF) AND intervention terms (CBA or RFA). The search terms were translated into Japanese when we searched the Ichushi‐Web database. Search strategies and keywords are shown in the Table S1. The scope of each search strategy is defined and reported in accordance with the *Population, Intervention, Comparison, Outcomes and Study* (PICOS) criteria contained in the Table S2. Although the systematic review included observational studies, the meta‐analysis only included randomized controlled trials (RCTs) and propensity score matching (PSM) cohort studies to minimize the effects of confounding.

A data extraction table was developed in Microsoft Excel to integrate data from included studies. General information regarding the identification of publication, such as author, title, year of publication, and study design were extracted. In addition, data on sample size, patient characteristics, treatment arm characteristics, and outcomes were also documented. Clinical effectiveness was assessed by recurrence of AF.

The quality assessment and risk of bias for each study was assessed using the NICE checklist for RCTs and the Downs and Black checklist for non‐RCTs.^16^, ^17^

### 2.2 Meta‐analysis

Risk ratio (RR) and hazard ratio (HR) with 95% confidence intervals (CIs) of AF recurrence were calculated. Studies were included in the meta‐analysis after considering the heterogeneity of the studies according to patient characteristics, intervention methods, and outcome definitions for each target population. Studies with a follow‐up period of 6 months or longer were included in the meta‐analysis. The data for RFA were pooled regardless of whether catheters incorporated 3D mapping or CF features in a base‐case analysis; however, a sensitivity analysis was performed by dividing data into CF‐RFA and non‐CF‐RFA group.

Both fixed and random effects models were applied and *p* < .05 was considered statistically significant. Heterogeneity among the included studies was analyzed using Cochran's *Q* test and an inconsistency index (*I* ^2^). The pooled results are displayed using forest plots. The statistical analysis was performed by R version 4.0.4.

### 2.3 Cost‐effectiveness analysis

#### 2.3.1 Overview

Based on the results of the meta‐analysis, the cost‐effectiveness of CBA versus RFA was assessed by cost minimization analysis (CMA). The target population for this analysis was patients with PAF who have previously failed one or more AADs and would receive ablative treatment for the first time for rhythm‐control purposes. Based on epidemiological studies, the starting age for the analysis population was 64.6 years and the proportion of males was 67%.^18^ The analysis was conducted from the Japanese public healthcare payer's perspective, estimating the direct medical costs over a lifetime. Future costs were discounted at a rate of 2% per year.

#### 2.3.2 Model structure

**FIGURE 1:**
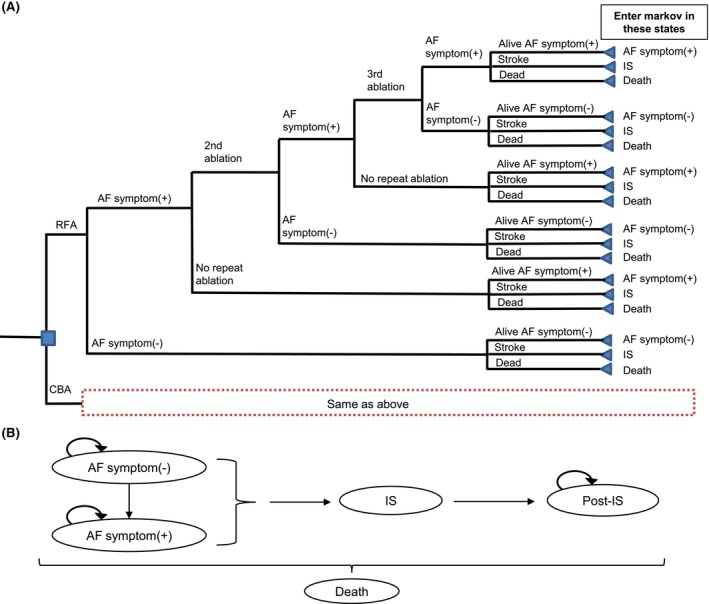
Model structure: (A) decision tree model, (B) Markov model. The occurrence of a postoperative complications is considered at the time of each ablation. Patients receiving a 3rd ablation are entered in the Markov model in year 3, and all other patients in year 2. AF, atrial fibrillation; CBA, cryoballoon ablation; IS, ischemic stroke; RFA, radiofrequency ablation.

The analytical model was built based on a previous cost‐effectiveness analysis of ablation therapies.^19^ The model consists of two parts: a decision tree to capture short‐term clinical outcomes (up to 2 years) and a Markov model to extrapolate lifetime clinical outcomes in a 1‐year cycle (Figure 1).

Patients with PAF enter the decision tree having undergone CBA or RFA. The decision tree reflects the period patients receive the ablation therapy and establishes whether patients are free of AF symptoms after ablation. The decision tree included four possible events: ischemic stroke (IS), AF symptoms, free from AF symptoms and death. Following ablation and AF symptom recurrence, a proportion would receive a repeat ablation. A maximum of two repeat ablations would be performed, with the second and third ablations to be performed at 6‐ and 18‐months post‐model entry, respectively. Repeat ablation was performed with a constant probability of CBA or RFA, the percentage of which was based on real‐world data from claims data (Medical Data Vision Co., Ltd.).^20^ For each ablation, postoperative complications were considered to include esophageal injury, cardiac tamponade, pulmonary vein stenosis, persistent diaphragmatic nerve palsy, vascular complications, and groin complications.

Patients who received a third ablation were entered into the Markov model in the third year, and all other patients were entered in the second year. Patients entered into the Markov model according to the health state at the end of the decision tree model as follows: those patients alive and free of AF symptoms entered the ‘free from AF symptoms (AF symptom (−))’ state, those alive and with AF symptom recurrence entered the ‘AF symptom (AF symptom (+))’ state, and finally those who had experienced a stroke whether or not they had AF symptoms, entered the ‘IS’ state. After entering the Markov model, patients in the AF symptom (−) state had a chance of reverting back to the AF symptom (+) state, having an ischemic stroke or dying. Patients in the AF symptom (+) state had a chance in each cycle of having an ischemic stroke or dying. Once AF symptoms recurred in the Markov model, they would no longer undergo ablative procedures. Patients who developed IS and survived for 1 year were moved to Post IS. The model does not take repeated IS into account.

#### 2.3.3 Transition probabilities

Each parameter of the transition probability was set based on published studies (Table 1). The results of the meta‐analysis show no significant difference in AF recurrence between CBA and RFA; therefore, the risk of AF recurrence after CBA was assumed to be the same as that after RFA. The risk of AF recurrence after the first ablation procedure and the proportion of patients having repeat ablation were defined based on Japanese epidemiological study data.^18^, ^21^ The risk of AF recurrence after the second ablation in patients who experienced AF recurrence following the first ablation was set to a higher probability than that at the first ablation (RR: 1.61).^19^ The risk of postoperative complications following ablation was set for each ablation procedure in accordance with the previous study.^19^ The stroke risk did not differ between patients with and without AF symptoms and was based on epidemiological studies.^19^, ^21^ Taking into account death from any cause, the mortality in the general population was used for AF symptoms (+) and (−), and mortality according to the disease condition obtained from the literature was used for IS and post IS.^22^, ^24^

**TABLE 1:** Model inputs.

	Value	DSA range		Source
		Lower	Upper	
Initial cohort settings				
Starting age^b^	64.6	63.9	65.3	[18]
Proportion of males^b^	67%	63.80%	70.20%	[18]
*Probabilities* (*decision tree*)				
AF recurrence after ablation (RFA/CBA)				
First ablation^a^	26.80%	21.40%	32.20%	[21]
Re‐ablation (RR)^a^	1.61	1.29	1.93	[19]
Re‐ablation rates after recurrent AF (RFA/CBA)				
2nd ablation^b^	78%	73.50%	82.50%	[18]
3rd ablation^b^	37%	21.40%	52.60%	[18]
Proportion of 2nd ablation				
RFA (1st ablation) to RFA (2nd ablation)^b^	98.30%	97.80%	98.80%	Claims data
CBA (1st ablation) to RFA (2nd ablation)^b^	96.40%	95.20%	97.60%	Claims data
Proportion of 3rd ablation				
RFA (2nd ablation) to RFA (3rd ablation)^b^	97.90%	96.10%	99.70%	Claims data
CBA (2nd ablation) to RFA (3rd ablation)^b^	100.00%	‐	‐	Claims data
*Serious adverse event risk*				
Oesophageal injury				
RFA/CBA^a^	0.50%	0.40%	0.60%	[19]
Cardiac tamponade				
RFA^a^	1%	0.80%	1.20%	[19]
CBA^a^	0.40%	0.30%	0.50%	[19]
Pulmonary vein stenosis				
RFA/CBA^a^	1%	0.80%	1.20%	[19]
Persistent diaphragmatic nerve palsy				
CBA^a^	1%	0.80%	1.20%	[19]
Vascular complications				
RFA/CBA^a^	2%	1.60%	2.40%	[19]
Groin complications				
RFA/CBA^a^	1%	0.80%	1.20%	[19]
Incidence of IS^a^ (RFA/CBA)	0.70%	0.60%	0.80%	[19]
Mortality^a^ (RFA/CBA)	1.20%	1.00%	1.40%	[19]
*Probabilities* (*Markov*)				
AF recurrence				
First and 2nd year in Markov^a^	7.70%	6.20%	9.20%	[21]
There after^a^	4.10%	3.30%	4.90%	[21]
Incidence of IS (AF symptom (+/−))^a^	0.24%	0.20%	0.30%	[21]
IS mortality				
Within 28 days (Fatal IS)^a^	7.30%	5.80%	8.80%	[22]
Within 1 year^a^ ^,^ ^c^	12.70%	10.20%	15.20%	[22]
Post IS (per year)^a^	8.50%	6.80%	10.20%	[22]
*Costs*				
Intervention costs (¥/event)				
RFA^b^	2 256 516	2 247 957	2 265 075	Claims data
CBA^b^	2 609 223	2 600 718	2 617 728	Claims data
Serious adverse event costs (¥/event)				
Oesophageal injury^b^	3 261 455	2 067 117	4 455 793	Claims data
Cardiac tamponade^b^	160 598	157 987	163 211	Claims data
Pulmonary vein stenosis^b^	1 679 342	1 152 122	2 206 562	Claims data
Persistent diaphragmatic nerve palsy^a^	15 230	12 184	18 276	Claims data
Vascular complications^b^	107 065	105 325	108 807	Claims data
Groin complications^b^	107 065	105 325	108 807	Claims data
Non‐fatal IS^b^	1 127 624	862 765	1 392 483	Claims data
Fatal IS (within 28 days)^b^	2 429 749	179 672	4 679 826	Claims data
Management costs (¥/year)				
AF symptom (+)^a^ ^,^ ^d^	172 780	138 224	207 336	Claims data
AF symptom (−)^b^ ^,^ ^d^	36 373	33 975	38 771	Claims data
Post IS^b^ ^,^ ^e^	67 486	21 579	113 393	Claims data
Discount rate (per year)	2%	0%	4%	[23]

Abbreviations: AF, atrial fibrillation; CBA, Cryoballoon ablation; CI, confidence interval; DSA, deterministic sensitivity analysis; IS, ischemic stroke; RFA, Radiofrequency ablation; RR, Relative risk.

^a^ Assume ±20% for DSA.

^b^ Parameters for sensitivity analysis were set with 95% CI.

^c^ Mortality among surviving cases at 28 days.

^d^ The costs covered were the drug cost of anticoagulant and antiarrhythmic drugs, tests, and visit fee in the outpatient department of internal medicine and cardiology.

^e^ Outpatient medical costs covered drug costs for antiarrhythmic drugs and anticoagulants, visit fee, and rehabilitation costs.

#### 2.3.4 Costs

Cost parameters were calculated using claims data for patients diagnosed with ‘PAF (ICD10 code: I480)’ and who underwent CBA (11 783 cases, mean age 67.1 ± 11.0, male%: 65.3%) or RFA (23 094 cases, mean age 67.9 ± 10.6, male%: 65.6%) on or after April 1, 2018.

To calculate the cost of each ablation procedure, the unit cost of hospitalization for patients undergoing CBA or RFA without postoperative complications was calculated. The prescription costs of anticoagulants in the 4 weeks before and 6 weeks after the date of admission and the cost of AADs in the 3 months after the date of admission were also calculated and included in the cost of the ablation procedure.^19^ Among the postoperative complications, “esophageal injury” and “pulmonary vein stenosis” were assumed to require rehospitalization, and the unit cost of hospitalization was calculated for patients who developed these complications after ablation.^19^ “Vascular complications,” “groin complications,” and “cardiac tamponade” were assumed to occur during hospitalization for ablation procedures. The costs were calculated assuming that these complications would require extended hospital stays: 2 extra days for “vascular complications,” 2 extra days for “groin complications,” and 3 extra days for “cardiac tamponade.”^19^ The cost of “persistent diaphragmatic nerve palsy” was calculated assuming that a CT scan and outpatient visit were required.^19^

A patient without a prescription for AADs at 1 year after ablation in the database was assumed to be in the AF symptom (−) state in the Markov model, and the administrative cost for the patient was used as the state cost of the AF symptom (−) state. Assuming that 67% of patients with symptomatic AF were prescribed AADs, the administrative cost for a patient in the AF symptom (+) state in the Markov model was calculated by weighting the cost for a patient receiving AADs and a patient not receiving AADs at 1 year after ablation in the database.^19^

The cost for IS was calculated based on patients who were diagnosed as having cerebral infarction (ICD‐10 Code: I63.X) after ablation, for fatal and non‐fatal cases. The cost of fatal IS was calculated with the hospitalization cost at the time of IS event for fatal cases. The hospitalization cost at the time of IS event and outpatient medical cost for 1 year post event was included in the cost of non‐fatal cases. In addition, the outpatient medical cost for the second year starting from 1 year post IS event was also calculated as the Post IS cost. Table 1 shows the cost parameters used for the analysis.

#### 2.3.5 Sensitivity analysis

A sensitivity analysis was performed to examine the stability and robustness of the results. In the deterministic sensitivity analysis, the discount rate was varied from 0% to 4% and other model inputs were varied by 95% CIs where available, or by ±20% of the base‐case value when CIs were not publicly available (Table 1).

## 3 RESULTS

### 3.1 Results of literature search for systematic review and meta‐analysis

The selection procedure with flow diagram for the included studies is shown in the Figure S1. Initially, 612 potentially relevant articles were identified in the preliminary literature search, of which 51 studies were eligible to be included in this systematic review. The meta‐analysis included only 12 RCTs and six propensity score matching (PSM) cohort studies. Of the 18 studies included in the meta‐analysis, eight studies included CF‐ RFA, six studies included non‐CF‐RFA, and in four studies it was not possible to determine the type of RFA. Two studies were conducted in Japan.

A summary of study characteristics is provided in Table 2.

**TABLE 2:** Baseline characteristics of the involved studies.

Study	Design	Country	Sample size	Mean age	PAF	Intervention (sample size)	Comparison (sample size)	Follow‐up time
Theis et al., 2022^25^	RCT	Germany	150	CBA = 61.62 RFA = 66.11	CBA = 100% RFA = 100%	CBA (*n* = 75)	CF RFA (*n* = 75)	12 months
Pak et al., 2021^26^	RCT	Korea	314	CBA = 60.8 RFA = 59	CBA = 100% RFA = 100%	CBA (*n* = 156)	Non‐CF RFA (*n* = 158)	9.8 months (mean)
Wang et al., 2021^27^	PSM cohort studies	China	192	CBA = 57.67 RFA = 57.59	CBA = 100% RFA = 100%	CBA (*n* = 96)	Non‐CF RFA (*n* = 96)	3 years
Andrade et al., 2019^28^	RCT	Canada	353	CBA 4 = 59.6 CBA 2 = 58.2 RFA = 58.6	CBA 4 = 94.8% CBA 2 = 97.4% RFA = 91.3%	CBA (*n* = 231)	CF RFA (*n* = 115)	12 months
Bin Waleed et al., 2019^29^	RCT	China	58	CBA = 61.2 RFA = 62.4	CBA = 100% RFA = 100%	CBA (*n* = 29)	CF RFA (*n* = 29)	6 months
Chang et al., 2019^30^	PSM cohort studies	Taiwan	138	CBA = 58.2 RFA = 56.3	CBA = 100% RFA = 100%	CBA (*n* = 69)	Non‐CF RFA (*n* = 69)	11.3 months (mean)
Giannopoulos et al., 2019^31^	RCT	Greece	120	CBA = 61 RFA = 58	CBA = 100% RFA = 100%	CBA (*n* = 80)	CF RFA (*n* = 40)	6 months
Ikenouchi et al., 2019^13^	PSM cohort studies	Japan	198	CBA = 77.7 RFA = 77.6	CBA = 83% RFA = 83%	CBA (*n* = 99)^a^	RFA (*n* = 99)^a^	12 months
Tokuda et al., 2019^14^	PSM cohort studies	Japan	460	CBA = 58.9 RFA = 58.7	CBA = 100% RFA = 100%	CBA (*n* = 230)	RFA (*n* = 230)	12 months
You et al., 2019^32^	RCT	China	210	CBA = 59.4 CBA 3D = 60.2 RFA = 57.7	CBA = 100% CBA 3D = 100% RFA = 100%	CBA (*n* = 140)	RFA (*n* = 70)	12 months
Buist et al., 2018^33^	RCT	Netherlands	269	CBA = 59.7 RFA = 58.2	CBA = 88.0% RFA = 82.4%	CBA (*n* = 133)^b^	CF RFA (*n* = 136)^b^	389 days (median)
Davtyan et al., 2018^34^	RCT	Russia	89	CBA = 57.6 RFA = 55.6	CBA = 100% RFA = 100%	CBA (*n* = 45)	Non‐CF RFA (*n* = 44)	12 months
Gunawardene et al., 2018^35^	RCT	Germany	60	CBA = 62 RFA = 57.4	CBA = 100% RFA = 100%	CBA (*n* = 30)	CF RFA (*n* = 30)	309.7 days (mean)
Matta et al., 2018^36^	PSM cohort studies	Italy	92	CBA = 59 RFA = 59	CBA = 100% RFA = 100%	CBA (*n* = 46)	CF RFA (*n* = 46)	12 months
Kuck et al., 2016^37^	RCT	Europe	750	CBA = 59.9 RFA = 60.1	CBA = 100% RFA = 100%	CBA (*n* = 374)	CF RFA (*n* = 376)	1.5 years (mean)
Hunter et al., 2015^38^	RCT	UK	237	CBA = 56 RFA = 61	CBA = 100% RFA = 100%	CBA (*n* = 78)	Non‐CF RFA (*n* = 77)	12 months
Knecht et al., 2014^39^	PSM cohort studies	Switzerland	142	CBA = 58.6 RFA = 57.8	CBA = 100% RFA = 100%	CBA (*n* = 71)	RFA (*n* = 71)	28 months (mean)
Perez et al., 2014^40^	RCT	Spain	50	CBA = 58 RFA = 56	CBA = 100% RFA = 100%	CBA (*n* = 25)	Non‐CF RFA (*n* = 25)	12 months

Abbreviations: CBA, cryoballoon ablation; CF, contact force; PAF, paroxysmal atrial fibrillation; PSM, propensity score matching; RCT, randomized clinical trial; RFA, radiofrequency ablation.

^a^ The meta‐analysis used the results of a subgroup analysis of patients with PAF (CBA: *n* = 82, RFA: *n* = 82).

^b^ The meta‐analysis used the results of a subgroup analysis of patients with PAF (CBA: *n* = 117, RFA: *n* = 112).

The results and summary of risk of bias assessment for included studies are summarized in the Table S3. There were no studies with a significantly high bias risk.

### 3.2 Results of meta‐analysis

**FIGURE 2:**
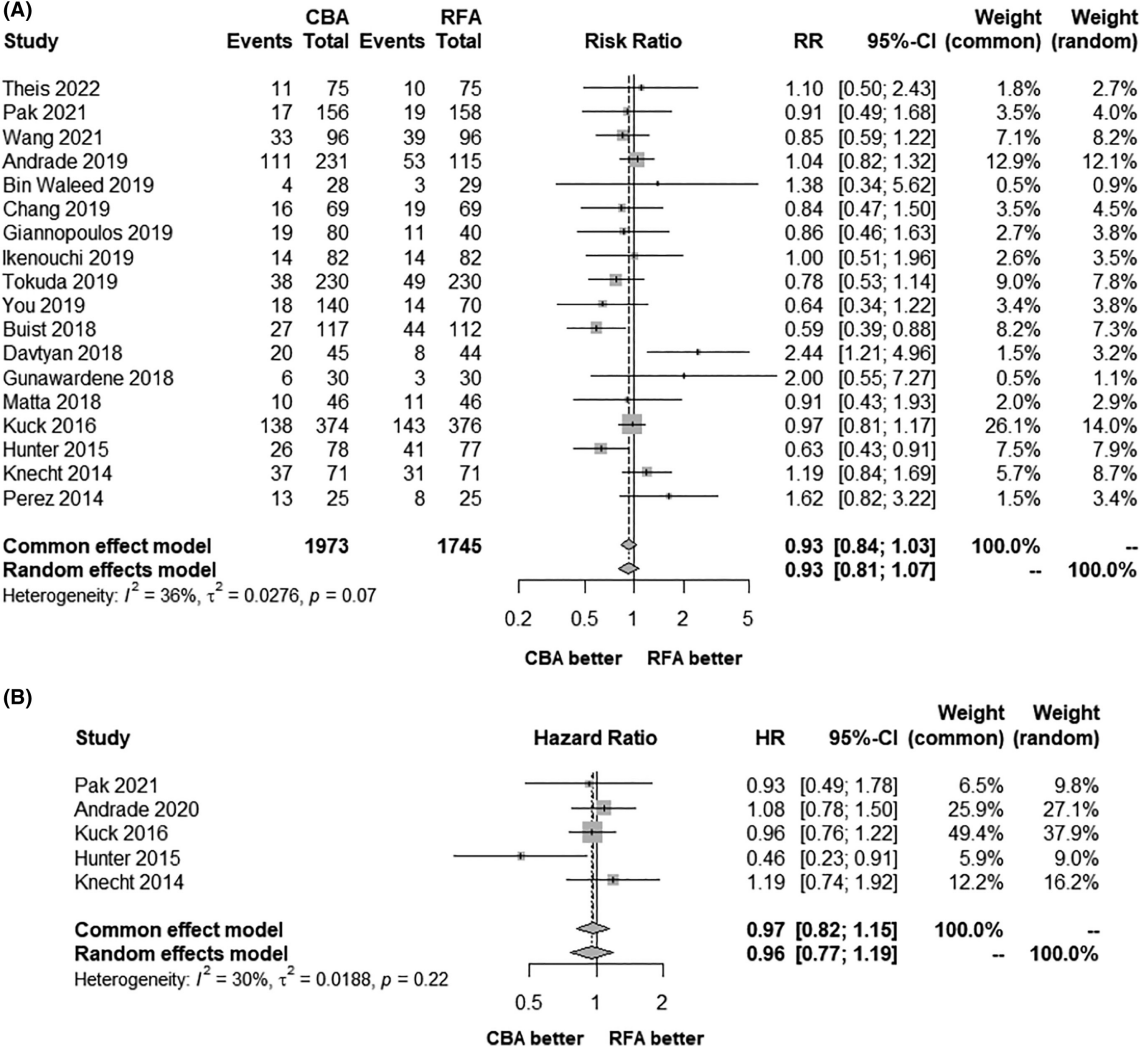
Forest plots of AF recurrence: (A) Risk ratio; (B) Hazard ratio. AF, atrial fibrillation; CBA, cryoballoon ablation; CI, confidence interval; HR, hazard ratio; RFA, radiofrequency ablation, RR, risk ratio.

**FIGURE 3:**
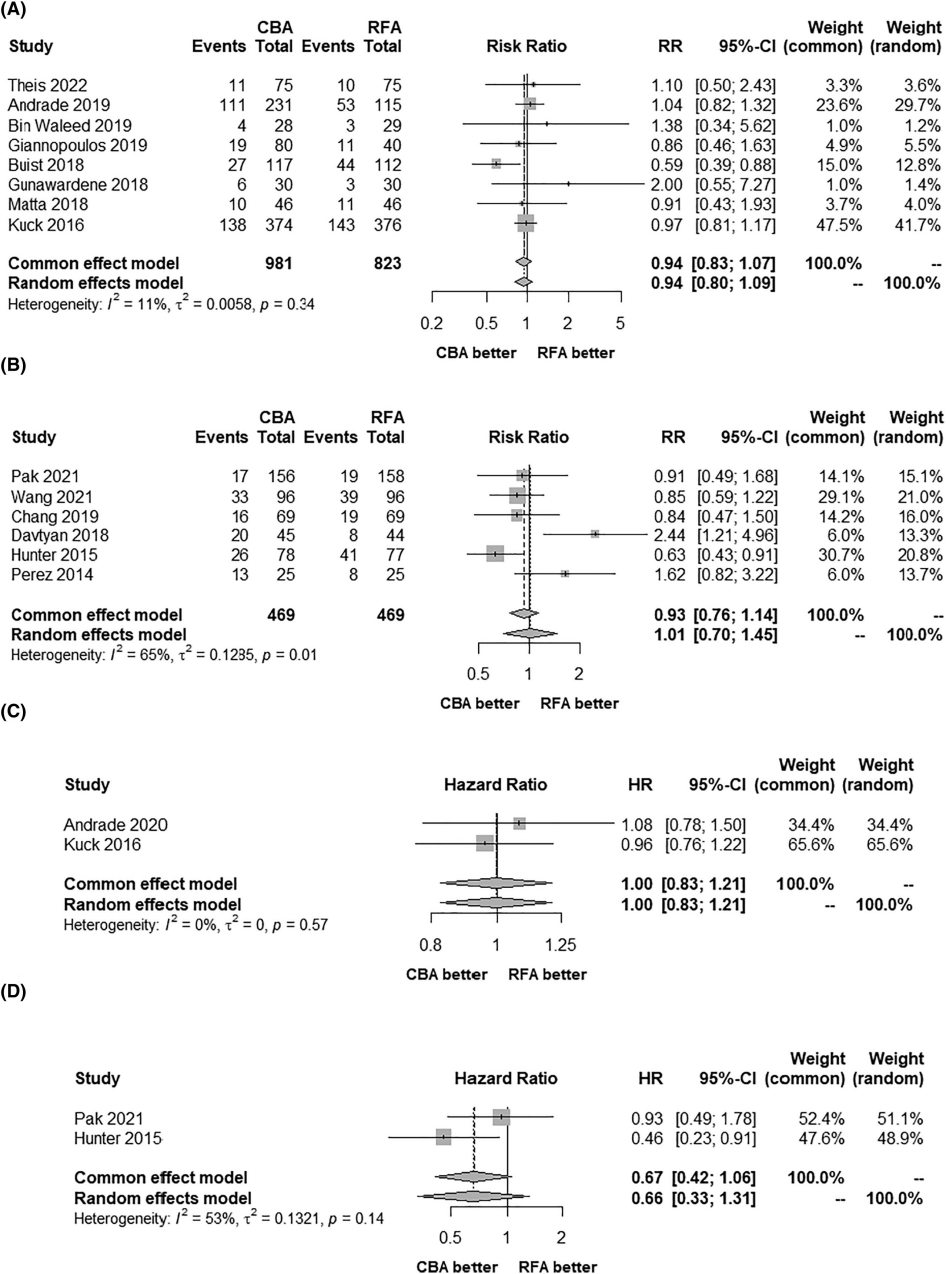
Forest plots of AF recurrence in sensitivity analysis: (A) CF‐RFA versus CBA (Risk ratio); (B) non‐CF‐RFA versus CBA (Risk ratio); (C) CF‐RFA versus CBA (Hazard ratio); (D) non‐CF‐RFA versus CBA (Hazard ratio). AF, atrial fibrillation; CBA, cryoballoon ablation; CF, contact force; CI, confidence interval; HR, hazard ratio; RFA, radiofrequency ablation, RR, risk ratio.

In the random effects models, AF recurrence after catheter ablation was slightly lower in patients referred for CBA than in those referred for RFA, with an integrated RR of 0.93 (95% CI 0.81–1.07) and an integrated HR of 0.96 (95% CI 0.77–1.19), but no significant difference was found. The results in the fixed effects models were similar (Figure 2). When the data were further divided into the CF‐RFA and non‐CF‐RFA groups, the results of each analysis indicated no statistically significant difference between CBA and RFA (Figure 3).

### 3.3 Results of cost‐minimization analysis

Cost minimization analysis was conducted to compare the medical costs between CBA and RFA because there were no significant differences in the risk of AF recurrence between CBA and RFA in the meta‐analysis. In the base‐case analysis, the estimated medical costs for CBA and RFA were JPY 4 858 544 (USD 32 390, USD 1 = JPY 150) and JPY 4 505 255 (USD 30 035), respectively.^41^ The cost savings for RFA was JPY 353 289 (USD 2355) compared with CBA. The costs related to postoperative complications were slightly lower for CBA than for RFA (JPY 46 268 [USD 308] vs. JPY 47 082 [USD 314]), while the costs associated with performing the ablation were higher for CBA than for RFA (JPY 3 157 643 [USD 21 051] vs. JPY 2 803 539 [USD 18 690]; Table 3).

**TABLE 3:** Results of cost‐minimalization analysis.

Strategy	RFA	CBA	Difference
Total	¥4 505 255	¥4 858 544	¥−353 289
Intervention costs	¥2 803 539	¥3 157 643	¥−354 103
Serious adverse event costs	¥47 082	¥46 268	¥814
Markov state costs			
AF symptom (−)	¥323 372	¥323 372	¥0
AF symptom (+)	¥1 255 734	¥1 255 734	¥0
Fatal‐IS	¥7687	¥7687	¥0
Non‐fatal IS	¥45 300	¥45 300	¥0
Post IS	¥22 541	¥22 541	¥0

Abbreviations: AF, atrial fibrillation; CBA, cryoballoon ablation; IS, ischemic stroke; RFA, radiofrequency ablation.

**FIGURE 4:**
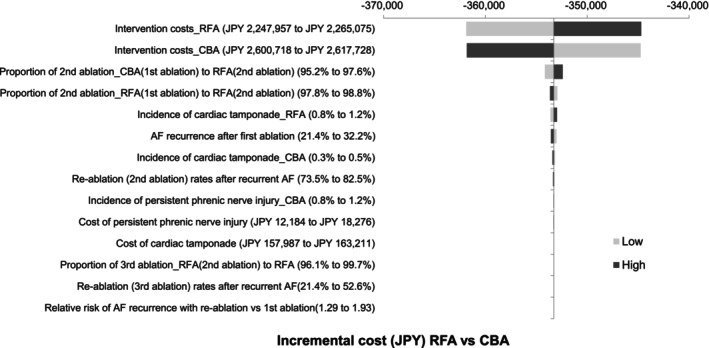
Tornado diagram for deterministic sensitivity analysis of cost‐minimization analysis. AF, atrial fibrillation; CBA, cryoballoon ablation; RFA, radiofrequency ablation; JPY, Japanese yen.

Results of the deterministic sensitivity analysis are displayed in a tornado diagram (Figure 4). Although the intervention costs of ablation had a large impact on the cost difference, RFA was cost saving compared with CBA in all of the ranges examined.

## 4 DISCUSSION

In this study, a meta‐analysis was conducted based on RCTs and PSM cohort studies that directly compared CBA and RFA. While RCTs are less likely to be affected by confounding factors and are suitable for the evaluation of effectiveness, in techniques such as ablation where effectiveness is affected by the skill level of individual physicians and the learning curve, the evidence from clinical research conducted in clinical settings also provides useful information. The PSM method has advantages, including achieving effects similar to randomization, balancing inter‐group confounders, and minimizing of inter‐group differences. Therefore, data from the PSM cohort studies were included in this meta‐analysis in addition to those from RCTs to assure the quality of evidence.

The results of the meta‐analysis show that both strategies were similar in terms of treatment effectiveness for PAF. There were no significant differences in the RR and HR of AF recurrence after catheter ablation between patients who underwent either CBA or RFA. The analysis performed by dividing RFA into CF‐RFA and non‐CF‐RFA groups showed similar trends as in the base‐case analysis. Meta‐analyses and network meta‐analysis have been conducted to assess the treatment effects of CBA and RFA, and many of the evaluation results demonstrated that the treatment effects of CBA are equivalent to those of RFA, indicating that our results are consistent with the results of the previous studies.^8^, ^9^, ^10^, ^11^, ^12^ Conversely, the meta‐analysis by Fortuni et al reported the superiority of CBA over RFA in terms of treatment effects.^7^ The type of RFA was not specified in the meta‐analysis by Fortuni et al., and its evaluation is based on data that included data from cohort studies. In contrast, the majority of studies that did not find a significant difference in treatment outcomes between CBA and RFA used data for CF‐RFA only, or data from RCTs only; therefore, the difference in the types of studies included in the analysis may have led to different results.

There have been overseas studies on the cost‐effectiveness of CBA compared with RFA, and cost utility analyses were performed in many of these studies.^42^, ^43^, ^44^ While the results of the cost‐effectiveness analyses could not be compared because they vary depending on the healthcare environment in each country, the results of the analyses varied from study to study. Murray et al. in the UK and Ming et al. in China reported that CBA was cost‐effective, whereas Darvish et al. in Iran reported that it was not. Although the previous studies used a variety of clinical evidence, these analyses used different AF recurrence rates between the two ablation techniques. In our study, CMA was conducted to assess the cost‐effectiveness of CBA, considering that the treatment effects of CBA are similar to those of RFA, based on the meta‐analysis results of our study, as well as the data from meta‐analyses and network meta‐analysis of the previous studies.^8^, ^9^, ^10^, ^11^, ^12^

In the CMA, in addition to AF recurrence rates, re‐ablation rates, incidence of IS, and mortality were set to be the same in both groups. A study assessing payer costs following CBA or RFA of PAF in the randomized FIRE AND ICE trial reported lower costs for CBA than RFA due to reduced repeat ablations and readmissions.^45^ On the other hand, the meta‐analysis by Murry et al. reported that repeat ablation procedures were not significant, and other previous meta‐analyses found no evidence of risk reduction with CBA for repeat ablation or readmission rates.^7^, ^8^, ^9^, ^10^, ^11^ In the absence of sufficient evidence of differences in repeat ablation and readmission, we assumed that the risk of these events was the same for RFA and CBA.

The results of CMA showed that the postoperative complication‐associated costs were slightly higher in the RFA than in the CBA, while the intervention costs were lower in the RFA, resulting in cost saving for RFA. The reduction in the medical cost for RFA was estimated to be JPY 353 289 (USD 2355) in our analysis. The reimbursement price for ablation catheters (highest class price at 2022) in Japan is JPY 649 000 (USD 4327) for CBA and JPY 395 000 (USD 2633) for RFA, with a difference of JPY 254 000 (USD 1693), indicating that the above cost reduction was greater than the difference in the device reimbursement price. There are cases in clinical practice in which RFA is also performed in patients who were to undergo CBA because the device does not come in close contact with the ablation site due to its shape, leading to insufficient ablation, and there is a possibility that such cases of CBA in combination with RFA may have contributed to a cost increase. In calculating the claims data, cases in which both RFA and CBA catheters were used during a single hospitalization were counted as cases in which CBA was performed and its total one hospitalization cost was calculated. In some countries other than Japan, touch‐up RFA is rarely performed after CBA because the intervention costs are reimbursed for a whole procedure, whereas in Japan, touch‐up RFA is more often performed after CBA partly because each device is reimbursed, not procedure.

Because the characteristics of postoperative complications differ between the procedures, the types and incidence of postoperative complications were defined separately for each procedure in the analysis; however, the difference in the cost incurred for postoperative complications was insignificant and had a negligible effect on analysis results.^19^

In this study, since it was suggested that the treatment effects of CBA are similar to those of RFA, a CMA was conducted, which demonstrated cost reduction in the case where RFA was used. However, some advantages of CBA, such as a shorter duration of procedure and less pain reported by patients, are not captured in the analysis because of the difficulty in incorporating these factors into a cost‐effectiveness analysis. Therefore, it is important to select a treatment option according to the patient's condition and other relevant factors.

Furthermore, this study has some limitations. First, this meta‐analysis included RCTs and PSM cohort studies, and in these studies, the evaluation period and the proportion of patients with comorbidities differed from one study to another, which may have been factors that could have confounded the results. Second, a sensitivity analysis was conducted by dividing RFA into CF‐RFA and non‐CF‐RFA group; however, not all retrieved papers were included because we were unable to identify the type of ablation. Third, although parameters that reflect the circumstances in Japan were selected as much as possible for the CMA, because of insufficient local evidence on the risk for postoperative complications associated with CBA and RFA, the parameters adopted in the analyses performed in other countries were used. The sensitivity analysis has demonstrated, however, that the effect of the risk for complications on the results of this analysis is insignificant. Finally, the analysis used hospital‐based claims data to estimate the costs, and there was a paucity of data on patients who died of stroke and patients who developed pulmonary vein stenosis, a complication of ablation, which leaves some uncertainty regarding the calculation results. Furthermore, the databases were on a hospital basis, if patients visited other clinics and hospitals for the purpose of management of post‐ablation or post IS, such data will not be reflected in the calculation, which may lead to an underestimation of the calculated costs. However, the costs will not affect the difference between CBA and RFA because we are using transitional probabilities common to both CBA and RFA; therefore, the cost saving results for RFA remain unchanged.

## 5 CONCLUSION

The meta‐analysis conducted in this study did not show any difference in the risk for AF recurrence between CBA and RFA, indicating that our results are consistent with those of previous studies. While the ablation procedure should be selected for each patient based on patient and procedure characteristics, our study indicated that as compared to CBA, RFA might be a cost‐saving procedure for the Japanese healthcare system.

## CONFLICT OF INTEREST STATEMENT

Yoshimi Nitta and Michiko Nishimura are current employees of Abbott Medical Japan LLC. Hideotoshi Shibahara is current employee of CRECON Medical Assessment Inc. CRECON Medical Assessment Inc. was paid from Abbott Medical Japan LLC to conduct analyses for the study. Teiichi Yamane received honoraria from Johnson & Johnson K.K., Medtronic Japan, consultant fees from Abbott Medical Japan LLC and BEG Co., Ltd.; and research grants from Japan Lifeline Co., Ltd.

## DECLARATIONS

*Approval of the research protocol*: N/A. *Informed Consent*: N/A. *Registry and the Registration No*.: N/A. *Animal Studies*: N/A.

## Supporting information


Data S1.
