# Supplementary material for: A meta‐analysis and cost‐minimization analysis of cryoballoon ablation versus radiofrequency ablation for paroxysmal atrial fibrillation
Source: J Arrhythm. 2024 Jun 9;40(4):802–14. doi: 10.1002/joa3.13055 (PMC11317739; doi:10.1002/joa3.13055)
Supplement: Supplementary file 1 — Data S1. [file JOA3-40-802-s001.docx]

Supplementary Materials:

Supplementary Table 1. Detailed search strategy.

1. MEDLINE

Dates searched：January 27, 2022

|  | | # | search terms | Results |
| --- | --- | --- | --- | --- |
| disease | PAF | 1 | PAF [tiab] or (paroxysmal [tiab] and "atrial fibrillation" [tiab]) | 20,621 |
|  |  | 2 | paroxysmal [tiab] and "atrial fibrillation" [MeSH Terms] | 7,503 |
|  |  | 3 | #1 or #2 | 20,894 |
| intervention | ablation | 4 | cryo*[tiab] or "Cryosurgery"[MeSH Terms] | 104,709 |
|  |  | 5 | ((radiofrequenc* [tiab] or "radio frequenc*" [tiab] or RF [tiab]) and (therap* [tiab] or surg* [tiab] or procedure* [tiab])) or "Radiofrequency Ablation"[MeSH Terms] or "point by point" [tiab] | 52,969 |
|  |  | 6 | #4 and #5 | 3,215 |
| ALL | | 7 | #3 and #6 | 511 |
| Study type | | 8 | #7 and (casereports[Filter] OR comment[Filter] OR editorial[Filter] OR letter[Filter] OR review[Filter]) | 80 |
|  |  | 9 | #7 Filters: Other Animals | 3 |
|  |  | 10 | #7 Filters:Humans | 463 |
|  |  | 11 | #9 not #10 | 1 |
|  |  | 12 | #7 not (#8 or #11) | 430 |

1. EMBASE

Dates searched：January 28, 2022

|  | | # | search terms | Results |
| --- | --- | --- | --- | --- |
| disease | PAF | 1 | paf:ti,ab | 16,466 |
|  |  | 2 | 'paroxysmal':ti,ab AND 'atrial fibrillation':ti,ab | 19,341 |
|  |  | 3 | paroxysmal:ti,ab AND 'atrial fibrillation'/exp | 18,840 |
|  |  | 4 | #1 OR #2 OR #3 | 33,196 |
| intervention | ablation | 5 | cryo*:ti,ab OR 'cryosurgery'/exp | 136,933 |
|  |  | 6 | radiofrequenc*:ti,ab OR 'radio frequenc*':ti,ab OR rf:ti,ab | 109,986 |
|  |  | 7 | therap*:ti,ab OR surg*:ti,ab OR procedure*:ti,ab | 7,859,127 |
|  |  | 8 | 'radiofrequency ablation'/exp OR 'point by point':ti,ab | 40,578 |
|  |  | 9 | #6 AND #7 OR #8 | 68,760 |
|  |  | 10 | #5 AND #9 | 4,709 |
| ALL | | 11 | #4 AND #10 | 936 |
| Study type | | 12 | #11 AND ([article]/lim OR [article in press]/lim) AND [embase]/lim | 294 |
|  |  | 13 | 'animal'/exp NOT 'human'/exp | 5,725,479 |
|  |  | 14 | #12 NOT #13 | 294 |

1. CENTRAL

Dates searched：January 28, 2022

|  | | # | search terms | Results |
| --- | --- | --- | --- | --- |
| disease | PAF | 1 | MeSH descriptor: [Atrial Fibrillation] explode all trees | 5,017 |
|  |  | 2 | 'atrial fibrillation':ti,ab | 12,722 |
|  |  | 3 | paroxysmal:ti,ab | 3,274 |
|  |  | 4 | PAF:ti,ab | 837 |
|  |  | 5 | (#1 or #2) and #3 | 2,137 |
|  |  | 6 | #4 or #5 | 2,605 |
| intervention | ablation | 7 | cryo*:ti,ab | 5,093 |
|  |  | 8 | MeSH descriptor: [Cryosurgery] explode all trees | 377 |
|  |  | 9 | #7 or #8 | 5,138 |
|  |  | 10 | MeSH descriptor: [Radiofrequency Ablation] explode all trees | 1,641 |
|  |  | 11 | radiofrequenc*:ti,ab or 'radio frequenc*':ti,ab or RF:ti,ab | 7,082 |
|  |  | 12 | therap*:ti,ab or surg*:ti,ab or procedure*:ti,ab | 654,903 |
|  |  | 13 | 'point by point':ti,ab | 70,763 |
|  |  | 14 | #10 or (#11 and #12) or #13 | 75,513 |
|  |  | 15 | #9 and #14 | 567 |
| ALL | | 16 | #6 and #15 | 135 |
|  |  | 17 | #6 and #15 in Trials | 135 |

1. Ichushi-Web

Dates searched：January 31, 2022

|  | | # | search terms | Results |
| --- | --- | --- | --- | --- |
| disease | PAF | 1 | 発作性[paroxysmal]/TA and (心房細動[Atrial Fibrillation] /TH or 心房細動[Atrial Fibrillation] /TA) | 4,989 |
| intervention | ablation | 2 | クライオ[cryo] /TA or cryo/TA | 5,271 |
|  |  | 3 | RF/TA | 73,950 |
|  |  | 4 | 高周波アブレーション[radiofrequency ablation]/TA or カテーテルアブレーション[catheter ablation]/TH or ラジオ波焼灼術[radiofrequency ablation]/TH | 30,656 |
|  |  | 5 | #3 or #4 | 100,313 |
|  |  | 6 | #2 and #5 | 631 |
| ALL | | 7 | #1 and #6 | 145 |
| Study type | | 8 | (#7) and ((PT=症例報告・事例除く [Excluding case reports and case studies]) AND (PT=原著論文[original articles only])) | 40 |

Supplementary Table 2. PICOS for inclusion and exclusion criteria for systematic review.

|  | Inclusion Criteria | Exclusion Criteria |
| --- | --- | --- |
| Patients | - Patients with paroxysmal AF who have previously failed one or more AAD and would receive ablative treatment for the first time for rhythm-control purposes | - Patients with AF due to severe valvular disease - Patients aged under 18 |
| Intervention | - CBA | - |
| Comparator | - RFA (Point-by-point method) | - |
| Outcome | - AF recurrence | - |
| Study type | - RCT with head-to-head comparison - Non RCT study with head-to-head comparison - PSM cohort studies with head-to-head comparison | - Single arm studies - Case reports - In vitro studies |
| Restrictions | - Full-text published manuscripts in English or Japanese - Year limitation: no limit | - Conference abstracts - Editorials - Reviews |

AAD, Antiarrhythmic drug; AF, atrial fibrillation; CBA, Cryoballoon ablation; PSM, propensity score matching; RCT, Randomized clinical trial; RFA, Radiofrequency ablation.

Supplementary Figure 1. Flow diagram of study selection for systematic review and meta-analysis.

Identification

Screening

Eligibility

Included

Excluded (n=556)

Reasons for exclusion

- Language (n=12)

- Population (n=40)

- Intervention (n=103)

- Study design (n=268)

- Article type (n=88)

- Outcomes (n=45)

Full-text articles assessed for eligibility

(n=56)

Records identified from MEDLINE

(n=437)

Records after duplicates removed

(n=612)

Records identified from EMBASE

(n=296)

Records identified from CENTRAL

(n=137)

Records identified from Ichushi Web

(n=40)

Studies included in qualitative synthesis

(n=51)

Excluded (n=5)

Reasons for exclusion

- Intervention (n=1)

- Outcomes (n=1)

- Sub-study of the involved studies (n=3)

Studies included in meta-analysis

(n=18)

Excluded (n=33)

Reasons for exclusion

- Short observation period of study (<6 months)

(n=1)

- Combined outcome of AF recurrence and

complication (n=1)

- Not RCTs or PSM cohort studies (n=31)

Supplementary Table 3. Risk of bias assessment for included studies in the meta-analysis.

A. RCT (Assessed using NICE checklist)

|  | Theis et al., 2022^1^ | Pak et al., 2021^2^ | Andrade et al., 2019^3^ | Bin Waleed et al., 2019^4^ | Giannopoulos et al., 2019^5^ | You et al., 2019^6^ | Buist et al., 2018^7^ | Davtyan et al., 2018^8^ | Gunawardene et al., 2018^9^ | Kuck et al., 2016^10^ | Hunter et al., 2015^11^ | Perez et al., 2014^12^ |
| --- | --- | --- | --- | --- | --- | --- | --- | --- | --- | --- | --- | --- |
| Was the randomisation method adequate? | not clear | yes | yes | not clear | not clear | yes | not clear | not clear | not clear | not clear | not clear | yes |
| Was the allocation adequately concealed? | not clear | not clear | no | not clear | no | not clear | not clear | not clear | not clear | not clear | not clear | not clear |
| Were the groups similar at the outset of the study in terms of prognostic factors, for example severity of disease? | yes | yes | yes | yes | yes | yes | yes | yes | yes | yes | yes | yes |
| Were the care providers, participants and outcome assessors blind to treatment allocation? If any of these people were not blind to treatment allocation, what might be the likely impact on the risk of bias (for each outcome)? | not clear | yes | yes | not clear | yes | not clear | not clear | not clear | not clear | no | no | not clear |
| Were there any unexpected imbalances in drop-outs between groups? If so, were they explained or adjusted for? | not clear | no | no | not clear | not clear | not clear | not clear | no | no | no | no | no |
| Is there any evidence to suggest that the authors measured more outcomes than they reported? | no | no | no | no | no | no | no | no | no | no | no | no |
| Did the analysis include an intention-to-treat analysis? If so, was this appropriate and were appropriate methods used to account for missing data? | yes | yes | yes | yes | yes | yes | yes | yes | yes | yes | yes | yes |

B. PSM cohort study (Assessed using Downs and Black checklist)

|  | Wang et al., 2021^13^ | Chang et al., 2019^14^ | Ikenouchi et al., 2019^15^ | Tokuda et al., 2019^16^ | Matta et al., 2018^17^ | Knecht et al., 2014^18^ |
| --- | --- | --- | --- | --- | --- | --- |
| Is the hypothesis/aim/objective of the study clearly described? | yes | yes | yes | yes | yes | yes |
| Are the main outcomes to be measured clearly described in the Introduction or Methods section? | yes | yes | yes | yes | yes | yes |
| Are the characteristics of the patients included in the study clearly described? | yes | yes | yes | yes | yes | yes |
| Are the interventions of interest clearly described? | yes | yes | yes | yes | yes | yes |
| Are the distributions of principal confounders in each group of subjects to be compared clearly described? | yes | yes | yes | yes | yes | yes |
| Are the main findings of the study clearly described? | yes | yes | yes | yes | yes | yes |
| Does the study provide estimates of the random variability in the data for the main outcomes? | yes | yes | yes | yes | yes | yes |
| Have all important adverse events that may be a consequence of the intervention been reported? | no | yes | yes | yes | yes | yes |
| Have the characteristics of patients lost to follow-up been described? | no | no | no | no | no | no |
| Have actual probability values been reported (e.g., 0.035 rather than <0.05) for the main outcomes except where the probability value is less than 0.001? | yes | yes | yes | yes | yes | yes |
| Were the subjects asked to participate in the study representative of the entire population from which they were recruited? | yes | yes | yes | yes | yes | yes |
| Were those subjects who were prepared to participate representative of the entire population from which they were recruited? | yes | yes | yes | yes | yes | yes |
| Were the staff, places, and facilities where the patients were treated, representative of the treatment the majority of patients receive? | yes | yes | yes | yes | yes | yes |
| Was an attempt made to blind study subjects to the intervention they have received? | no | no | no | no | no | no |
| Was an attempt made to blind those measuring the main outcomes of the intervention? | no | no | no | no | no | no |
| If any of the results of the study were based on “data dredging”, was this made clear? | unable to determine | unable to determine | unable to determine | unable to determine | unable to determine | unable to determine |
| In trials and cohort studies, do the analyses adjust for different lengths of follow-up of patients, or in case-control studies, is the time period between the intervention and outcome the same for cases and controls? | yes | yes | yes | yes | yes | yes |
| Were the statistical tests used to assess the main outcomes appropriate? | yes | yes | yes | yes | yes | yes |
| Was compliance with the intervention/s reliable? | yes | yes | yes | yes | yes | yes |
| Were the main outcome measures used accurate (valid and reliable)? | yes | yes | yes | yes | yes | yes |
| Were the patients in different intervention groups (trials and cohort studies) or were the cases and controls (case-control studies) recruited from the same population? | yes | yes | yes | yes | yes | yes |
| Were study subjects in different intervention groups (trials and cohort studies) or were the cases and controls (case-control studies) recruited over the same period of time? | yes | yes | yes | yes | yes | yes |
| Were study subjects randomised to intervention groups? | not applicable | not applicable | not applicable | not applicable | not applicable | not applicable |
| Was the randomised intervention assignment concealed from both patients and health care staff until recruitment was complete and irrevocable? | not applicable | not applicable | not applicable | not applicable | not applicable | not applicable |
| Was there adequate adjustment for confounding in the analyses from which the main findings were drawn? | yes | yes | yes | yes | yes | yes |
| Were losses of patients to follow-up taken into account? | unable to determine | unable to determine | unable to determine | unable to determine | unable to determine | unable to determine |

**REFERENCES**

1. Theis C, Kaiser B, Kaesemann P, Hui F, Pirozzolo G, Bekeredjian R, et al. Pulmonary vein isolation using cryoballoon ablation versus RF ablation using ablation index following the CLOSE protocol: A prospective randomized trial. J Cardiovasc Electrophysiol. 2022;33(5):866-73.
2. Pak HN, Park JW, Yang SY, Kim TH, Uhm JS, Joung B, et al. Cryoballoon Versus High-Power, Short-Duration Radiofrequency Ablation for Pulmonary Vein Isolation in Patients With Paroxysmal Atrial Fibrillation: A Single-Center, Prospective, Randomized Study. Circ Arrhythm Electrophysiol. 2021;14(9):e010040.
3. Andrade JG, Champagne J, Dubuc M, Deyell MW, Verma A, Macle L, et al. Cryoballoon or Radiofrequency Ablation for Atrial Fibrillation Assessed by Continuous Monitoring: A Randomized Clinical Trial. Circulation. 2019;140(22):1779-88.
4. Bin Waleed K, Yin X, Yang X, Dai B, Liu Y, Wang Z, et al. Short and long-term changes in platelet and inflammatory biomarkers after cryoballoon and radiofrequency ablation. Int J Cardiol. 2019;285:128-32.
5. Giannopoulos G, Kossyvakis C, Vrachatis D, Aggeli C, Tsitsinakis G, Letsas K, et al. Effect of cryoballoon and radiofrequency ablation for pulmonary vein isolation on left atrial function in patients with nonvalvular paroxysmal atrial fibrillation: A prospective randomized study (Cryo-LAEF study). J Cardiovasc Electrophysiol. 2019;30(7):991-8.
6. You L, Yao L, Zhou B, Jin L, Yin H, Wu J, et al. Effects of different ablation strategies on long-term left atrial function in patients with paroxysmal atrial fibrillation: a single-blind randomized controlled trial. Sci Rep. 2019;9(1):7695.
7. Buist TJ, Adiyaman A, Smit JJJ, Ramdat Misier AR, Elvan A. Arrhythmia-free survival and pulmonary vein reconnection patterns after second-generation cryoballoon and contact-force radiofrequency pulmonary vein isolation. Clin Res Cardiol. 2018;107(6):498-506.
8. Davtyan K, Shatakhtsyan V, Poghosyan H, Deev A, Tarasov A, Kharlap M, et al. Radiofrequency versus Cryoballoon Ablation of Atrial Fibrillation: An Evaluation Using ECG, Holter Monitoring, and Implantable Loop Recorders to Monitor Absolute and Clinical Effectiveness. Biomed Res Int. 2018;2018:3629384.
9. Gunawardene MA, Hoffmann BA, Schaeffer B, Chung DU, Moser J, Akbulak RO, et al. Influence of energy source on early atrial fibrillation recurrences: a comparison of cryoballoon vs. radiofrequency current energy ablation with the endpoint of unexcitability in pulmonary vein isolation. Europace. 2018;20(1):43-9.
10. Kuck KH, Brugada J, Fürnkranz A, Metzner A, Ouyang F, Chun KR, et al. Cryoballoon or Radiofrequency Ablation for Paroxysmal Atrial Fibrillation. N Engl J Med. 2016;374(23):2235-45.
11. Hunter RJ, Baker V, Finlay MC, Duncan ER, Lovell MJ, Tayebjee MH, et al. Point-by-Point Radiofrequency Ablation Versus the Cryoballoon or a Novel Combined Approach: A Randomized Trial Comparing 3 Methods of Pulmonary Vein Isolation for Paroxysmal Atrial Fibrillation (The Cryo Versus RF Trial). J Cardiovasc Electrophysiol. 2015;26(12):1307-14.
12. Pérez-Castellano N, Fernández-Cavazos R, Moreno J, Cañadas V, Conde A, González-Ferrer JJ, et al. The COR trial: a randomized study with continuous rhythm monitoring to compare the efficacy of cryoenergy and radiofrequency for pulmonary vein isolation. Heart Rhythm. 2014;11(1):8-14.
13. Wang X, Song B, Qiu C, Han Z, Wang X, Lu W, et al. The effect of left atrial remodeling after cryoballoon ablation and radiofrequency ablation for paroxysmal atrial fibrillation. Clin Cardiol. 2021;44(1):78-84.
14. Chang TY, Lo LW, Te ALD, Lin YJ, Chang SL, Hu YF, et al. The importance of extrapulmonary vein triggers and atypical atrial flutter in atrial fibrillation recurrence after cryoablation: Insights from repeat ablation procedures. J Cardiovasc Electrophysiol. 2019;30(1):16-24.
15. Ikenouchi T, Nitta J, Nitta G, Kato S, Iwasaki T, Murata K, et al. Propensity-matched comparison of cryoballoon and radiofrequency ablation for atrial fibrillation in elderly patients. Heart Rhythm. 2019;16(6):838-45.
16. Tokuda M, Yamashita S, Matsuo S, Kato M, Sato H, Oseto H, et al. Clinical significance of early recurrence of atrial fibrillation after cryoballoon vs. radiofrequency ablation-A propensity score matched analysis. PLoS One. 2019;14(7):e0219269.
17. Matta M, Anselmino M, Ferraris F, Scaglione M, Gaita F. Cryoballoon vs. radiofrequency contact force ablation for paroxysmal atrial fibrillation: a propensity score analysis. J Cardiovasc Med (Hagerstown). 2018;19(4):141-7.
18. Knecht S, Sticherling C, von Felten S, Conen D, Schaer B, Ammann P, et al. Long-term comparison of cryoballoon and radiofrequency ablation of paroxysmal atrial fibrillation: a propensity score matched analysis. Int J Cardiol. 2014;176(3):645-50.
